# Dosimetric Comparison of Hypofractionated Regimen in Breast Cancer Using Two Different Techniques: Intensity-Modulated Radiation Therapy (IMRT) and Volumetric-Modulated Arc Therapy (VMAT)

**DOI:** 10.7759/cureus.38045

**Published:** 2023-04-24

**Authors:** Pallav Prasun, Vipin Kharade, Vikas Pal, Manish Gupta, Saikat Das, Rajesh Pasricha

**Affiliations:** 1 Radiation Oncology, All India Institute of Medical Sciences, Bhopal, Bhopal, IND

**Keywords:** volumetric-modulated arc therapy, hypofractionated regimen, dosimetric analysis, intensity-modulated radiation therapy, breast cancer

## Abstract

Introduction: Breast cancer treated with adjuvant hypofractionation radiotherapy with two different techniques, i.e., volumetric-modulated arc therapy (VMAT) and intensity-modulated radiation therapy (IMRT) and their effects in terms of loco-regional control and adverse effects in terms of cutaneous, pulmonary, and cardiac outcomes are compared.

Materials and methods: This is a prospective non-randomized observational study. VMAT and IMRT plan for 30 breast cancer patients who were supposed to receive adjuvant radiotherapy were prepared using a hypofractionation schedule. The plans were dosimetrically evaluated.

Objective: Dosimetric comparative analysis of IMRT and VMAT in hypofractionated radiotherapy in breast cancer is done and tested whether VMAT has a dosimetric advantage over IMRT. These patients were recruited for a clinical assessment of toxicities. They were followed up for at least three months.

Result: On dosimetric analysis, planning target volume (PTV) coverage (PTV_ _V95_) of both VMAT (96.41 ± 1.31) and IMRT (96.63 ± 1.56) were similar with significantly lower monitor units required with VMAT plans (1,084.36 ± 270.82 vs 1,181.55 ± 244.50, p = 0.043). Clinically, all patients tolerated hypofractionation through VMAT (n = 8) and IMRT (n = 8) satisfactorily in the short term. No cardiotoxicity or appreciable falls in pulmonary function test parameters were observed. Acute radiation dermatitis poses challenges similar to standard fractionation or any other delivery technique.

Conclusion: PVT dose, homogeneity, and conformity indices were similar in both VMAT and IMRT groups. In VMAT, there was high-dose sparing of some critical organs like the heart and lungs at the cost of the low-dose baths to these organs. Increased risk of secondary cancer will require a decade-long follow-up study to indict the VMAT technique. As we move toward precision in oncology, “one-size-fits-all” can never be an acceptable dictum. Each patient is unique and therefore we must offer, and the patient must “choose wisely.”

## Introduction

According to World Health Organization (WHO) estimates for 2019, cancer ranks first or second leading cause of death in a population younger than 70 years in 112 of 183 countries and is third or fourth in a further 23 countries and inversely correlates with the Human Development Index (HDI) based on the United Nation's 2019 Human Development Report [1]. According to Global Cancer Observatory (Globocan) 2020 data, with an estimated 2.3 million new cases (11.7%), female breast cancer has become the most frequently diagnosed cancer, surpassing lung cancer narrowly. It is the fifth leading cause of cancer mortality worldwide, with 685,000 deaths annually [1]. In consonance with the observation that breast and cervical cancer burden transitioning countries more, in India, breast cancer is the most common cancer among females 26.3%, and overall, 13.5% as well. Even in terms of five-year prevalence and mortality, breast cancer ranks the highest. In light of these gloomy figures, it becomes imperative to develop a multi-disciplinary approach to holistically limit the socio-economic-psychological ill effects of this disease [2].

Management of breast cancer has evolved from the years of Halsted's highly mutilating radical mastectomy to the present-day multimodality approach comprising surgery, chemotherapy, and radiotherapy. Radiotherapy (RT) plays a major role in the treatment - both, for the loco-regional control of the disease and survival either after mastectomy or after Breast Conservation Surgery (BCS) especially when four to seven lymph nodes are positive for tumor cells. The main toxicities of adjuvant radiotherapy for breast cancer include cutaneous, pulmonary, and cardiac related. National Comprehensive Cancer Network (NCCN) accepted 45-50.4 Gray (Gy) in 25-28 fractions over five to 5.5 weeks as conventional schedule and 40-42.5 Gy in 15-16 fractions over three weeks as a hypofractionated schedule to standardize treatment regimens [3]. With mature evidence from randomized trials [4,5], hypofractionated whole-breast radiation (WBR) and hypofractionated post-mastectomy radiotherapy (PMRT) [6,7] have demonstrated equivalent rates of local-regional recurrence, disease-free survival, and overall survival as compared to conventional fractionation. Patient preferences, logistical issues, ﬁnancial considerations, advances in radiobiological knowledge allowing for a more specific estimation of equivalent dosing, and advances in the delivery of RT modulation that have resulted in substantially improved dose homogeneity in the target volume, have influenced the increased acceptance of hypofractionated schedules [7]. In pursuance of the emerging robust long-term evidence, the American Society for Therapeutic Radiology and Oncology (ASTRO) in its 2018 update, mandated hypofractionation for both post-BCS and post-mastectomy breast cancer patients.

Thus, unintended high radiation doses can be reduced in critical structures such as the skin, lungs, and heart, thus ensuring decreased early and late adverse effects. Furthermore, evidence abounds where intensity modulation of beams can give incremental benefit over three-dimensional conformal radiotherapy (3D-CRT) in terms of reducing toxicities [8]. If toxicity were not enough, the sloping surfaces of the chest wall pose a different sort of challenge in planning and delivery of radiation. Use of tangential fields with collimation and compensators have been traditionally used to reduce the volume of lungs in the radiation fields. However, the problem of junction matching between the anterior supraclavicular foss (SCF) field, and the bi-tangential field still poses the problem of resulting hot/cold spots. The use of an appropriately angled breast board gets rid of collimation as it elevates the thoracic cage so to make the chest wall horizontal [9].

RT planning has gradually progressed from two-dimensional (2D) to three-dimensional (3D) conformal therapy with forward-planned field-in-field to inverse-planned intensity-modulated radiation therapy (IMRT) and variations of IMRT of which volumetric-modulated arc therapy (VMAT) is common. This has been possible because of computed tomography (CT)-based contouring and electron-density-based planning. The focus is maximal target dose coverage and homogeneity with minimal dose to The Organs At Risk (OAR). Standard RT fields with standard regimens treated in the past had a very low risk of loco-regional recurrence so it is important to ensure that no significant changes in the field size or arrangement are used. This requires optimum delineation of target volumes and contouring of OARs taking into consideration anatomical variations in different sets of patients. Large inter and intra-observer variations in the volume of regional lymph nodes, breast, and chest wall have been observed. To minimize this, several guidelines have been proposed for target volume delineation in breasts by American, European, and Trans-Tasman radiation oncology societies [10-13].

IMRT uses inverse treatment planning most of the time; however, forward treatment planning has been utilized in the past. The quantity, orientations, and shape of the beams are used to estimate the radiation delivery profile in advanced treatment planning. Collimator-based IMRT makes use of moveable collimators to change the intensity of the beam over each field. Multi-leaf collimation (MLC) treatment uses static positions for the collimator leaves, whereas IMRT allows for dynamic mobility of the collimator leaves during each therapy session [14].

Many variations of IMRT have been developed to simplify the treatment. Various modalities of intensity modulation have already shown a relative advantage over each other depending on patient and tumor characteristics. One novel modification has been the adoption of the VMAT technique to further the options at radiation oncologists' hands. It is based on the simultaneous optimization of an MLC, gantry rotation, and dose rate giving greater target volume coverage and homogeneity when compared to its predecessor [15]. VMAT has consistently fared better than conventional tangential field techniques in reducing ipsilateral lung doses. Even though earlier VMAT techniques have been reported to increase the mean dose of the contralateral lung, contralateral breast, and heart, evidence has built over time to routinely employ it in clinical practice and explore whether the clinical outcome is congruent with its physics [16,17]. It additionally offsets few other disadvantages of IMRT such as increased treatment time, larger monitor units, leakage, and scatter radiation which have implications on repair and repopulation of tumor cells.

The present study aims to dosimetrically compare the VMAT technique with IMRT in delivering a hypofractionated regimen in breast cancer patients and study their effects in terms of loco-regional response and cutaneous, pulmonary, and cardiac adverse effects.

## Materials and methods

A prospective, single-center, non-randomized, observational study was conducted with a total of 30 patients enrolled for the study. The study was approved by Institutional Human Ethics Committee (IHEC) - Post-Graduate Research, All India Institute of Medical Sciences, Bhopal (IHEC-PGRMD056). All 30 patients aged 18-70 years included in the dosimetric study had 30 IMRT and 30 VMAT plans made and evaluated. Out of 30 patients included in the dosimetric study, only 16 patients who were treated with IMRT or VMAT with a hypofractionated regimen were included in the toxicity study. Histopathologically confirmed cases of breast cancer, Eastern Cooperative Oncology Group (ECOG) performance score of 0-2, post-operated cases of breast-conserving surgery or mastectomy, non-metastatic breast cancer (Tis -T4c pN0-3, M0), and minimum gap of two weeks from prior treatment modality (chemotherapy/surgery). All patients were included irrespective of targeted or hormonal therapy as this was a dosimetric study. Metastatic disease breast cancer, patients with immediate reconstruction, patients having already received breast/chest wall irradiation, patients with collagen vascular disease, forced expiratory volume in one second (FEV1) < 80%, and left ventricular ejection fraction (LVEF) < 45% on 2D-echo were excluded from the study.

Quality assurance

All the patients satisfying the inclusion criteria underwent routine evaluation and standard pre-radiotherapy work-up as per our department protocols were followed. It included thorough history and physical examination, routine baseline blood investigations, and histopathological review if needed. In addition, the patient underwent 2D echocardiography and a pulmonary function test (PFT). Patients were staged according to the American Joint Committee on Cancer (AJCC) staging system, 8th edition based on clinical and pathological data. The use of neoadjuvant or adjuvant or any ongoing targeted or hormonal therapy was noted.

**Figure 1:**
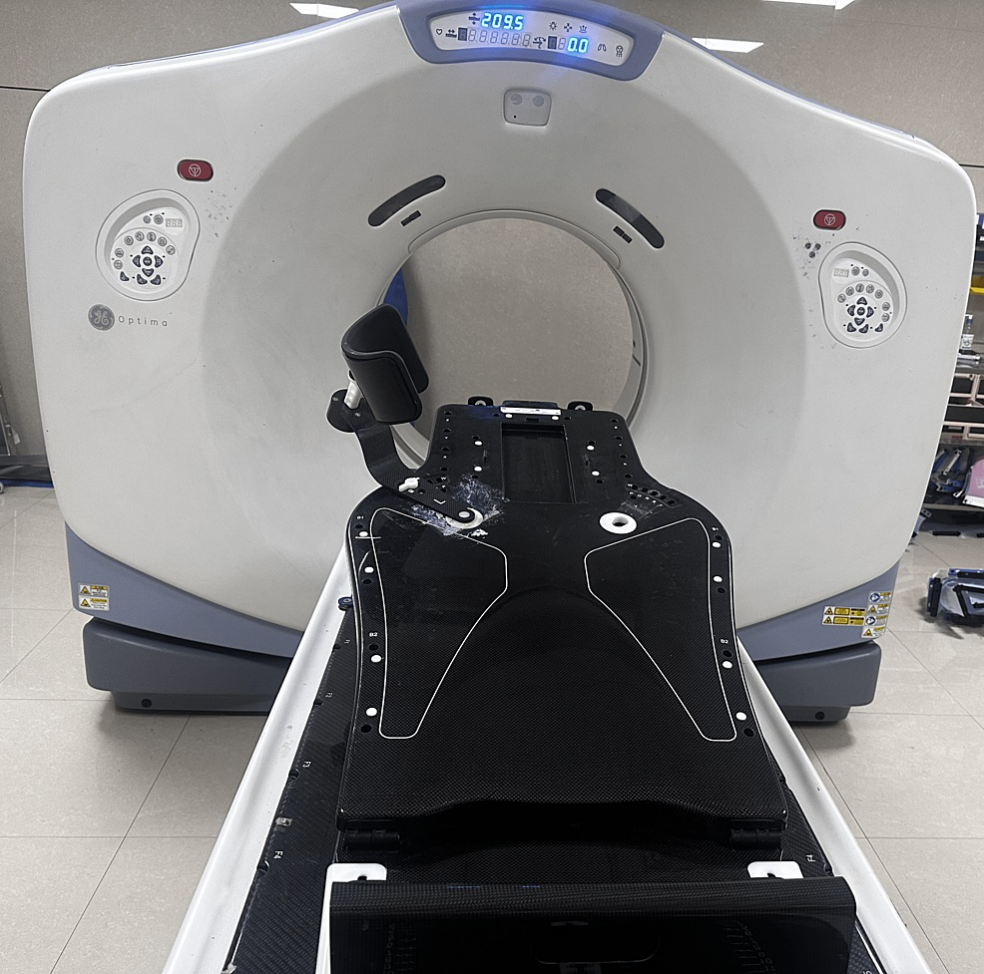
Breast Board

**Figure 2:**
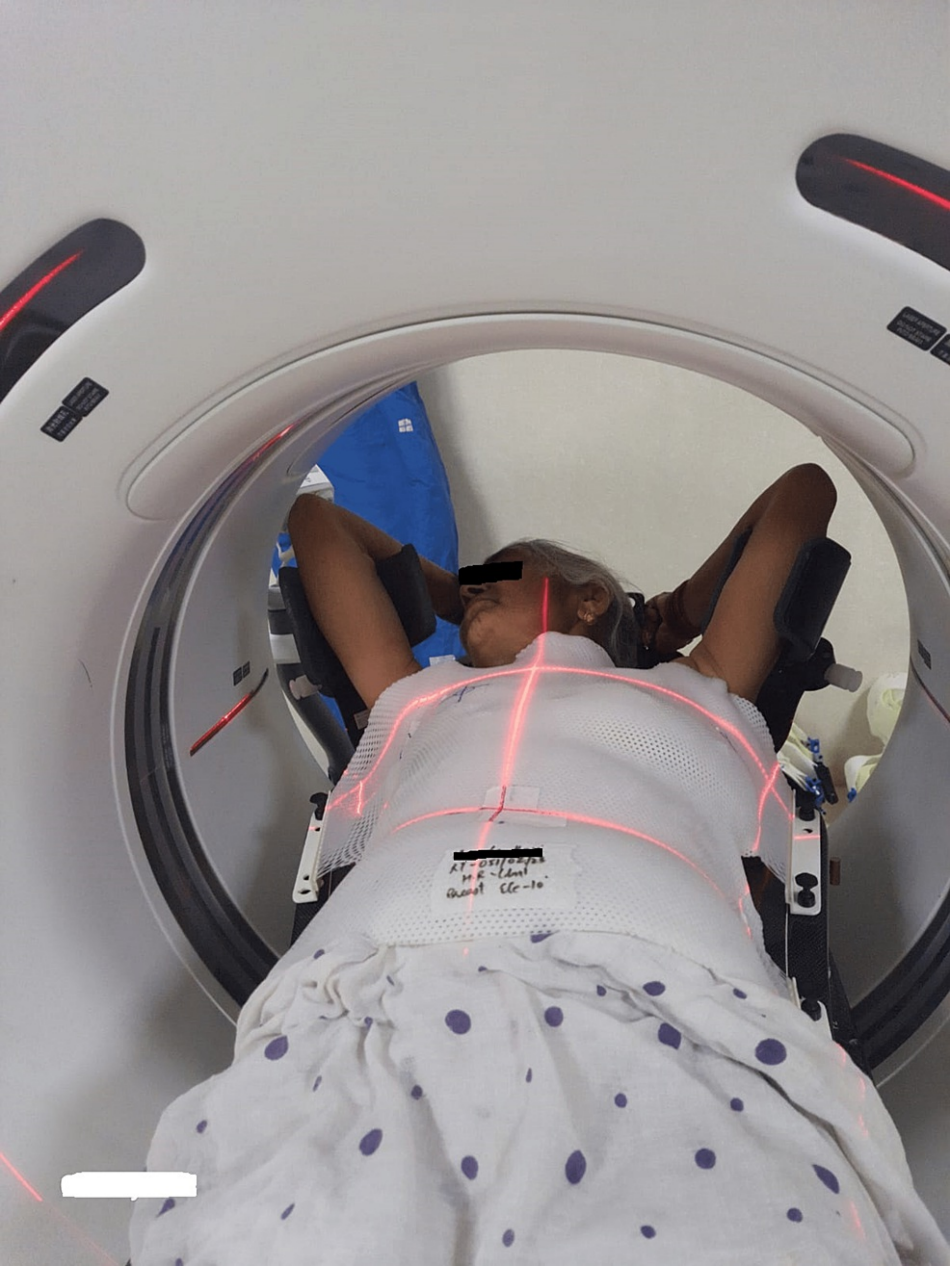
Computed tomography (CT) image acquisition The CT scan images acquired on Optima CT-580 RT (GE Healthcare, India)

After obtaining informed consent, the patients requiring radiotherapy were taken for mold room procedures, which consist of positioning the patient on a breast board (Figure 1) and preparing a thermoplastic cast which helps in immobilization and reproducibility of position during imaging and treatment. Additionally, a snugly yet comfortably fit cast helps in restricting breathing movement. Patients were asked to lie supine with their heads tilted in opposite directions to avoid the trachea from the direct radiation portal to the affected side. Patients were instructed to abduct and raise their arms above their heads. In this position, the mold for the patients was prepared and marked with identifiers. In cases where the thermoplastic cast was not used, vacuum cushions were fabricated. Patients were then repositioned on the CT machine couch. Radio-opaque markers identifiable on CT imaging were used to mark the mid-axillary fold (ipsilateral) for lateral margin, postoperative breast or chest wall scars, inferior margin (1.5 cm to 2.0 cm) of palpable breast tissue or opposite breast in MRM patients, medial margin at midline, superior margin at the base of clavicle and if present, drain sites also. Thermoplastic casts were replaced on the chest. External fiducial markers were applied to the thermoplastic cast at the center level of the radio opaque marks to aid treatment planning and repositioning. With the patient in the treatment position, CT images were acquired at 2.5- or 3-mm slice thickness from the angle of the mandible to the lower border of the L2 vertebra (Figure 2). Patients were instructed to relax and breathe normally. Provided the renal function was normal non-ionic intravenous contrast was given at 1-1.5 mL/kg to aid precise target delineation.

Volume delineation and dose prescription

**Figure 3:**
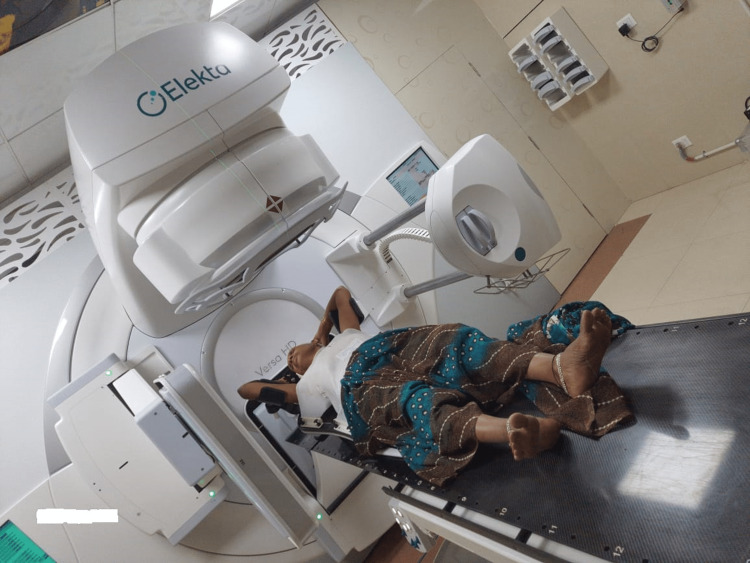
A typical radiotherapy treatment set-up on the linear accelerator

The CT scan images acquired on Optima CT-580 RT (GE Healthcare, India) were transferred to a radiation therapy contouring and planning workstation (MONACO treatment planning system (TPS), version 5.11.02), where target volumes were delineated as per Radiation Therapy Oncology Group (RTOG) guidelines for breast cancer radiotherapy on axial slices by a radiation oncologist [13]. After target volume delineation, the patient was taken for treatment on the linear accelerator (Figure 3).

Other than whole-breast or chest-wall depending upon the surgery performed, the axillary region, supraclavicular fossa, and internal mammary chain was included in the target volume where indicated. The OAR included ipsilateral and contralateral lungs, heart, left anterior descending (LAD) artery, opposite breast, spinal cord, esophagus, ipsilateral humeral head, and brachial plexus. For the dosimetric study, patients have prescribed a total dose of 40.05 Gy in 15 fractions. OAR constraints were protocol-specific and arrived after analyzing landmark hypofractionation trials and Quantitative Analyses of Normal Tissue Effects in the Clinic (QUANTEC) criteria [18]. Whether the patients were treated with standard or hypofractionation regimens using IMRT or VMAT was left to the oncologists’ discretion as this was a dosimetric observation study. Isocenter was placed at the center of the PTV.

IMRT Planning

Five to nine coplanar intensity modulated fields around the patient’s breast/chest wall were used. The IMRT plans were optimized to cover at least 95% of PTV by 95% of the prescribed dose while minimizing the dose to OARs as much as possible. Inverse planning optimizations were performed using Monaco TPS (version 5.11.02). Dose calculation is done with the Monte Carlo algorithm with a 3-mm grid resolution. The auto flash margin of 1.5 cm to PTV was used to extend the fluence outside the body contour. The treatment planning optimization process took tissue inhomogeneities into account. An optimization with as many iterations as required to achieve the planning goals was applied. They were followed by a semiautomatic segmentation. Segments with less than ≤2 Monitor Units (MUs) were removed from the plan.

VMAT Planning

Plans were generated with Monaco TPS (version 5.11.02) in which continuous gantry motion was modeled as several discrete angle segments and MLC apertures were progressively added throughout the optimization. MU per gantry angle will be optimized using a variable dose rate. The gantry incremental angle will be kept at 1 degree. The Monte Carlo dose calculation algorithm will be used with heterogeneity corrections and a grid size of 3 mm. The auto flash margin of 1.5 cm to PTV will be used to extend the fluence outside the body contour. Two dual arcs of 50-60 degrees. The collimator angle will be set to 0 degrees.

Plan Evaluation

**Figure 4:**
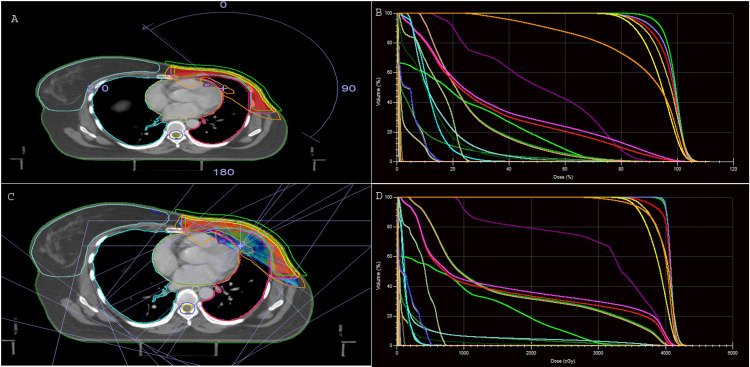
VMAT plan (A) and DVH (B); IMRT plan (C), and DVH (D) Abbreviation - Volumetric Modulated Arc Therapy (VMAT), Intensity-Modulated Radiation Therapy (IMRT), Dose-Volume Histogram (DVH)

Dose-volume histograms (DVH) were used to evaluate all plans. All the plans were approved by a consultant radiation oncologist after they were found satisfactory concerning PTV coverage and avoidance of OAR according to the protocol criteria. DVHs were used for evaluation and comparisons of dose to OARs and all the parameters required for the study. The VMAT and IMRT (Figures 4A-4D).

Plans were evaluated and compared for target coverage (V95 and D90); conformity index (CI), homogeneity index (HI); monitor units (MU); dose to ipsilateral lung (V20, V10, V5, and mean dose); dose to heart (V30, V25, V10, V2, and Dmean); dose to LAD; dose to contralateral breast (V2 and Dmean), the mean dose to the skin, mean dose to the esophagus, and mean dose to ipsilateral brachial plexus and humeral head. Target volume coverage and its conformity were evaluated using The International Commission on Radiation Units and Measurements (ICRU) 83 [19]​​​​​​.

Since toxicity evaluation in patients who went ahead to be treated with a hypofractionated regimen through IMRT or VMAT technique was part of the study, a thorough Quality assurance was done with PTW Octavius 4D Phantom. The mean difference between the prescribed and measured doses was assessed. Additionally, some of the radiotherapy treatment plans were collected and analyzed by the quality assurance team to ensure compliance with the protocol in terms of prescription point and dose homogeneity. A dose variation (in the PTV) between 95% and 107% of that at the reference point on the central axis was allowed in the protocol.

Verification and Treatment Delivery

For patients in toxicity study, after verification of treatment set up with cone beam CT images, necessary corrections were applied before treatment delivery. Treatment was done on Linac Accelerator (Elekta Versa HD). All patients were reviewed once a week for treatment. Due to logistical issues due to the COVID-19 situation, patients were reviewed anytime between the ninth and 15th-week post-treatment for acute reactions. Then between the 15th and 25th week post-radiotherapy, for morbidity assessment, the Common Terminology Criteria for Adverse Events (CTCAE) version, 5.0 was used for scoring skin toxicity. A radiological grading scale for radiation pneumonitis as proposed by Kouloulias et al. was used. Statistical data were generated for acute cutaneous, pulmonary, and cardiac toxicity (occurring within six months of RT). Other predictable complaints typical in a breast cancer patient like dysphagia, shoulder stiffness, hematological abnormalities, limb pain, and lymphedema were also noted. PFT, ECG, 2-D Echo, and routine blood investigations were repeated between the ninth and 15th week and/or between the 15th and 25th week for all patients. A CT thorax was also done during this period in our own department and reviewed with the radiologist.

Statistical analysis

This non-randomized prospective study was proposed to be a pilot study with some participants 60. Since the principal aim of this project is to find agreement trajectories between dosimetric comparisons, the sample size was tailored as per the distribution of variables (non-parametric/Poisson’s distribution). In keeping with the logistic issues, patient inflow, and COVID-19 situation, a sample size of 30 was achieved 16 of whom were subjects for a clinical study. The data were initially entered into Microsoft Excel from the customized master chart and then online statistical software GraphPad and Epi info were used for calculating the p-values. A comparison of mean dosimetry values between IMRT and VMAT was done using paired t-test. Association between two non-parametric variables was done using the Pearson Chi-square test. Correlation between two parametric variables within VMAT and IMRT was done using the Pearson Co-efficient of Correlation. A p-value of < 0.05 was taken as statistically significant. The final data were presented in the form of tables and graphs.

## Results

The mean demographic and tumor characteristics are shown in Table 1.

**Table 1:** Demographic and tumor characteristics Abbreviations: IDC - Invasive Duct Carcinoma Mucinous, ER - Estrogens Receptor, PR - Progesterone Receptor, Her2 - Human Epidermal growth factor Receptor 2

Characteristics	No. of patient n=30	Percentage, n (%)
Mean age	44.93 ± 9.34 (range: 29 to 64 years)	
Laterality		
Left side	20	66.7
Right side	10	33.3
Menopausal status		
Premenopausal status	15	50.0
Postmenopausal status	15	50.0
Carcinoma staging		
IA	2	6.7
IB	1	3.3
IIA	2	6.7
IIB	2	6.7
IIIA	8	26.7
IIIB	11	36.7
IIIC	4	13.3
Histopathology findings		
IDC	28	93.3
IDC, mucinous	1	3.3
Phylloides	1	3.3
Biological assessment		
ER receptor		
Positive	17	56.7
Negative	12	40
PR receptor		
Positive	16	53.3
Negative	13	43.3
Her2-neu receptor		
Positive	8	26.7
Negative	21	70.0
Surgery		
Breast conservative surgery	8	26.7
Modified radical mastectomy	22	73.3
Irradiation to supraclavicular fossa	26	86.7
Irradiation to internal mammary gland	15	50.0
Use of bolus	17	56.7

The mean planning target volume (PTV_V95) of VMAT was 96.41 ± 1.31 and for IMRT was 96.63 ± 1.56. The difference was found to be statistically not significant (P=0.481), showing a comparable mean PTV_V95. The mean PTV_D90 of VMAT was 39.92 ± 0.73 and for IMRT was 39.73 ± 0.53. The difference was found to be statistically not significant (P=0.217), showing a comparable mean PTV_D90. The mean MU/Fraction of VMAT was 1084.36 ± 270.82 and for IMRT was 1181.55 ± 244.50. The difference was found to be statistically significant (P=0.043), showing a significantly higher MU/fraction in IMRT patients compared to VMAT patients (Table 2).

**Table 2:** Comparison of planning target volume (PTV_V95, MU/fraction and PTV_D90) between VMAT, and IMRT Abbreviations: PTV-Planning target volume, PTV_V95 -PTV volume receiving 95% of the prescribed dose, MU-Monitor unit, PTV_D90-PTV doses covering 90% of volume, VMAT- Volume modulated arc therapy, IMRT- Intensity-modulated radiation therapy

Modality	No.	PTV_V95 [Mean ± SD]	MU/Fraction [Mean ± SD]	PTV_D90 [Mean ± SD]
VMAT	30	96.41 ± 1.31	1084.36 ± 270.82	39.92 ± 0.73
IMRT	30	96.63 ± 1.56	1181.55 ± 244.50	39.73 ± 0.53

The mean ipsilateral lung_Volume receiving 5 Gy (V5) of VMAT was 81.87 ± 14.64, and for IMRT was 72.83 ± 13.52. The difference was statistically significant (P=0.001), showing a significantly lower ipsilateral lung_V5 on IMRT compared to VMAT, and the mean contralateral lung_Dmean of VMAT was 5.35 ± 1.73 and for IMRT was 4.73 ± 2.62. The difference was statistically insignificant (P=0.181), showing a comparable contralateral lung_Dmean between the VMAT and IMRT (Table 3).

**Table 3:** Comparison of mean ± SD between VMAT and IMRT (n=30) Abbreviations: V5 - Volume receiving 5Gy, V30 - Volume receiving 30Gy, V2 - Volume receiving 2Gy, VMAT - Volume-modulated arc therapy, IMRT - Intensity-modulated radiation therapy, SD - Standard deviation

Parameter	VMAT	IMRT	P-value
Ipsilateral Lung_V5 .	87 ± 14.64	72.83 ± 13.52	0.001
Heart_V30	3.24 ± 2.53	8.49 ± 6.66	0.001
Heart_V2	98.86 ± 3.36	94.85 ± 9.24	0.013
Heart_Dmean	9.11 ± 2.30	11.29 ± 3.77	0.003
LAD_Dmean	14.81 ± 6.15	15.91 ± 7.15	0.183
Breast contralateral_V2	72.19 ± 18.26	54.89 ± 24.97	0.001
Esophagus_Dmean	11.86 ± 4.58	13.16 ± 5.28	0.046
Patients who had undergone left-sided irradiation			
Parameter	VMAT	IMRT	P value
Dose to Heart_V30	3.87 ± 2.17	11.27 ± 6.16	0.001
Dose to Heart_Dmean	9.41 ± 2.58	12.44 ± 3.59	0.001

The mean heart_Volume receiving 30Gy (V30) of VMAT was 3.24 ± 2.53 and for IMRT was 8.49 ± 6.66. The difference was statistically significant (P=0.001), showing a significantly higher heart_V30 in IMRT compared to the VMAT, The mean heart_Volume receiving 2Gy (V2) of VMAT was 98.86 ± 3.36, and for IMRT was 94.85 ± 9.24. The difference was statistically significant (P=0.013), showing a significantly lower Heart_V2 in IMRT compared to the VMAT and the mean Heart_Dmean of VMAT was 9.11 ± 2.30 and for IMRT was 11.29 ± 3.77. The difference was statistically significant (P=0.003), showing a significantly higher Heart_Dmean in IMRT compared to the VMAT.

For patients who had undergone left-sided irradiation, the mean dose to Heart_V30 of VMAT was 3.87 ± 2.17, and for IMRT was 11.27 ± 6.16. The difference was found to be statistically significant (P=0.001), showing a significantly higher dose of Heart_V30 in IMRT patients compared to VMAT patients. The mean dose to Heart_Dmean of VMAT was 9.41 ± 2.58, and for IMRT was 12.44 ± 3.59. The difference was found to be statistically significant (P=0.001), showing a significantly higher dose to Heart_Dmean in IMRT patients compared to VMAT patients.

The mean LAD_Dmean of VMAT was 14.81 ± 6.15 and for IMRT was 15.91 ± 7.15. The difference was found to be statistically not significant (P=0.183), showing a comparable mean LAD_Dmean between the two modalities, The mean breast contralateral_V2 of VMAT was 72.19 ± 18.26 and for IMRT was 54.89 ± 24.97. The difference was statistically significant (P=0.001), showing a significantly lower mean breast contralateral_V2 in IMRT compared to the VMAT.

## Discussion

Breast cancer has emerged as the most commonly diagnosed cancer worldwide and in India. Radiotherapy is integral to loco-regional control both in post-mastectomy and in patients having undergone breast-conserving surgery. Long-term results of the Ontario trial in Canada and START trials in the UK [20] have given confidence in using shorter schedules which have been pushed to the limit in FAST-FORWARD trials. From 2D to 3D conformity to modulation has led to the wide popularity of IMRT and VMAT as a modality of choice in delivering radiation. Even though dosimetric data for use of VMAT in breast cancer abound, clinical studies left more to be desired [21]. As the number of monitor units, beam on time, and treatment time are lower for VMAT as compared to IMRT, it is fast getting in vogue with most hospitals. In our present study, we made an endeavor to combine both dosimetric and clinical studies to explore whether the relative dosimetric advantage of newer techniques manifests clinically.

Thirty patients for whom dosimetric comparison was done had a mean age of about 45 years (range: 29-64 years) with half of them pre-menopausal. Many cancer registries in India have also warned that breast cancer occurs at a younger age [22]. The majority (22 out of 30) underwent a mastectomy and had their supraclavicular fossa irradiated (26 out of 30), which implies that breast cancer is aggressive in the younger age group. Moreover, 23 out of 30 (76%) were high-grade tumors. 50% of our patients were planned for internal mammary nodal irradiation. In a critical appraisal of VMAT in radiation therapy management of breast cancer, Luca Cozzi and colleagues found a general trend in the majority of the studies regarding VMAT [23]. It is good for optimal target coverage, homogeneity, and conformity with this technique. Sparing of ipsilateral OAR is equivalent and can be further improved [24]. Ashraf et al. found 3D-CRT to be better than IMRT in terms of PTV conformity index and sparing of critical structures in whole breast irradiation [25]. Popescu et al. found VMAT better in achieving similar PTV coverage and sparing OARs with fewer MUs and delivery time when compared with IMRT [26]. In our study, PTV_V95 mean values were similar for both IMRT and VMAT plans (96.63 ± 1.56 vs 96.41 ±1.31) but VMAT required significantly lesser MUs which manifests in lesser treatment time. It is cost-effective and patient friendly. It means less scatter radiation dose also. The mean MUs per fraction for VMAT in our study is 1,084.36 ± 270.82 whereas for IMRT it is 1,181.55 ± 244.50; with a p-value of 0.043, this difference is significant. We did not find any significant correlation between target volume and MUs per fraction. In our study, V30 and V25 mean doses were significantly lower (V30 mean, 3.24 ± 2.53 Gy) in the VMAT plan than IMRT (8.49 ± 6.66 Gy), with a p-value of 0.001. But expectedly, this advantage was a trade-off with an increased low-dose bath. Mean V2 was significantly lower with IMRT. Darby et al. concluded that there is no apparent threshold dose for developing long-term cardiotoxicity, which increased linearly at 7.4% per gray [27]. Dmean to heart in our study was about 23% lower for VMAT which was significant (p = 0.003). This improvement was further accentuated to about 32% (9.41 Gy with VMAT vs 12.44 Gy in IMRT) when we take only left-sided breast cancers into account (20 out of 30). In a comparative dosimetric study by Zhao et al, combining VMAT with DIBH could possibly further reduce the mean heart dose (MHD) by 2.9 Gy (1.5 Gy-4.3 Gy) [24]. Goldman et al investigated short-term radiation pneumonitis using changes in PFTs in patients of breast cancer in which regional nodal irradiation included internal mammary field found that if ipsilateral lung constraints of V20 < 30% were adhered to, there was a 6% incidence of symptomatic radiation pneumonitis [28]. In our study, both IMRT and VMAT plans narrowly missed the mark (30.68 ± 7.59 Gy for VMAT and 32.12 ± 4.94 Gy for IMRT) with insignificant difference between the two (p = 0.169). Half the patients in our study (15 out of 30) had IMN in the treatment field. Since the risk of acute and chronic RT-induced pulmonary morbidity is influenced by the irradiated lung volume, total dose, and dose per fraction [29], both VMAT and IMRT may be preferred. Again, where IMRT has a significant advantage over VMAT is the smaller volume with low-dose exposure. Even though no grade 3 or higher pneumonitis rates have been observed even when ipsilateral lung V5 was 100% [30], we must be wary of the fact that the latency of secondary cancers after RT is ≥ 10 years.

The contralateral breast received a significantly higher low dose in VMAT. Even though the mean dose in VMAT and IMRT were comparable (4.56 ± 1.44 and 4.66 ± 1.99 Gy, respectively), volume receiving 2% of the dose, mean V2 was 72.19 ± 18.26 in the case of VMAT compared to 54.89 ± 24.97 Gy in IMRT plans. This higher low-dose bath increases the risk of secondary cancer in long term. Lee et al compared IMRT, VMAT, and 3D-CRT measuring phantom doses with photoluminescent detectors and demonstrated that the lifetime attributable risk (LAR) of secondary malignancies for VMAT was lower than IMRT but higher than 3D-CRT [31]. Radiation dermatitis develops in a deterministic, dose-dependent manner with temporal predictability. In the plans, skin-sparing was not evident as the mean dose in both plans almost reached the target dose. For the toxicity study, we could manage only 16 patients, eight in each group. At the end of the week, one had developed mild erythema. While 50% of patients in the VMAT group did not develop hyperpigmentation, 87.5% (seven out of eight) in the IMRT group had hyperpigmentation. As has been adequately documented, skin reaction progressively worsens throughout the course of treatment [32]. By the end of radiation treatment, all the patients had developed at least grade 2 dermatitis. While none of the patients in the VMAT group had progressed to grade 3, one (12.5%) in IMRT had grade 3 confluent moist desquamation. On the first follow-up at about three months, re-epithelialization had mitigated the radiation damage in five out of eight patients in the VMAT group, and five out of eight (62.5%) patients in the IMRT group still had dry desquamation. Hyperpigmentation may persist for months as had been found by Buchholz et al. [33]. It is worth noting that of the 10 patients in which bolus was used, seven (70%) developed radiation fibrosis by the end of the study. Breast cancer patients less often complain of dysphagia in and after the second week of treatment. This is particularly true for left-sided irradiation due to the anatomy. On DVH analysis, the mean dose to the esophagus was significantly lower for VMAT than IMRT. If we analyze only the left-sided breast cancer plans, the mean dose to the esophagus expectedly increased for both, but it increased more for the IMRT plan. While it was 12.66 ± 4.43 Gy for VMAT, it was 14.39 ± 4.48 for IMRT and the 13.67% difference was significant (p = 0.028).

Even though breast plans never reach the tolerance dose of brachial plexus, after going through chemotherapeutic agents which may cause neuropathy and in patients in which supraclavicular fossa is also irradiated, the mean dose to ipsilateral brachial plexus must be limited. In our study, there was no significant difference noted and the mean dose was about 37 Gy in each. Clinically, limb pain is earlier manifested. On analyzing only, the patients who received supraclavicular fossa (26 out of 30) irradiation, the mean dose increased to about 42 Gy. Of the 16 patients in a clinical study, 14 had received SCF irradiation and eight of them mentioned limb pain during the course of the study. While only it was 28.6% (two out of seven) for IMRT-treated patients, the corresponding number was 85.6% (six out of seven) in the VMAT group. Even though the difference was significant (p = 0.031), the direct correlation may not be reached. Shoulder stiffness is another infrequent complaint. It may or may not be associated with lymphedema. It is mainly due to the dose to the humeral head. In our study, mean dose to the ipsilateral humeral head was 11.83 ± 6.71 Gy in the case of VMAT and 10.72 ± 5.09 Gy in IMRT with the difference being insignificant. Unlike pelvic irradiation, hematological toxicity is not a major concern in breast radiotherapy. But due to prior hemotoxic chemotherapy, there may be a depletion in blood counts. The worst hematological toxicity noted in our study was grade 2 with only one patient having her hemoglobin falling below 8 g/dL.

Limitation

This is a single-center study. A bigger study with large sample size and long follow-up will help to further understand the dosimetric and toxicity outcome.

## Conclusions

The choice of radiotherapy technique and optimum fractionation schedule is of paramount importance in breast cancer treatment. In our study, IMRT and VMAT plans were compared dosimetrically for the hypofractionation regimen. PTV dose, homogeneity, and conformity indices were similar in both groups. We also achieved a few expected results. There was high-dose sparing of some critical organs like the heart and lungs at the cost of the low-dose both to these organs. It is a necessary trade-off that fasts improving optimization techniques may offset. Increased risk of secondary cancer will require decades-long follow-up studies to indict the VMAT technique. As we move toward precision in oncology, “one-size-fits-all” can never be an acceptable dictum. Each patient is unique and therefore we must offer, and the patient must “choose wisely.” Clinically, our study could not appreciate remarkable improvements in toxicity profile. Of the results that we achieved, hypofractionation is well-tolerated in a heterogeneous group of patients who formed part of our study. More clinical studies with long-term follow-ups are needed to support our results and further assess clinical outcomes.
